# Clinical characteristics and outcomes of patients with recurrent status epilepticus episodes

**DOI:** 10.1186/s42466-023-00261-9

**Published:** 2023-07-13

**Authors:** Kristina Bauer, Felix Rosenow, Susanne Knake, Laurent M. Willems, Leena Kämppi, Adam Strzelczyk

**Affiliations:** 1 Epilepsy Center Frankfurt Rhine-Main and Department of Neurology, University Hospital and Goethe-University Frankfurt, Schleusenweg 2-16 (Haus 95), 60528 Frankfurt am Main, Germany; 2 LOEWE Center for Personalized Translational Epilepsy Research (CePTER), Goethe-University Frankfurt, Frankfurt am Main, Germany; 3 Epilepsy Center Hessen and Department of Neurology, Philipps-University Marburg, Marburg (Lahn), Germany; 4 Epilepsia Helsinki, European Reference Network EpiCARE, Department of Neurology, Helsinki University Hospital and University of Helsinki, Helsinki, Finland

**Keywords:** Epilepsy, Seizure, Recurrence, Recurrent episodes

## Abstract

**Background:**

Multiple studies have focused on medical and pharmacological treatments and outcome predictors of patients with status epilepticus (SE). However, a sufficient understanding of recurrent episodes of SE is lacking. Therefore, we reviewed recurrent SE episodes to investigate their clinical characteristics and outcomes in patients with relapses.

**Methods:**

In this retrospective, multicenter study, we reviewed recurrent SE patient data covering 2011 to 2017 from the university hospitals of Frankfurt and Marburg, Germany. Clinical characteristics and outcome variables were compared among the first and subsequent SE episodes using a standardized form for data collection.

**Results:**

We identified 120 recurrent SE episodes in 80 patients (10.2% of all 1177 episodes). The mean age at the first SE episode was 62.2 years (median 66.5; SD 19.3; range 21–91), and 42 of these patients were male (52.5%). A mean of 262.4 days passed between the first and the second episode. Tonic–clonic seizure semiology and a cerebrovascular disease etiology were predominant in initial and recurrent episodes. After subsequent episodes, patients showed increased disability as indicated by the modified Rankin Scale (mRS), and 9 out of 80 patients died during the second episode (11.3%). Increases in refractory and super-refractory SE (RSE and SRSE, respectively) were noted during the second episode, and the occurrence of a non-refractory SE (NRSE) during the first SE episode did not necessarily provide a protective marker for subsequent non-refractory episodes. An increase in the use of intravenous-available anti-seizure medication (ASM) was observed in the treatment of SE patients. Patients were discharged from hospital with a mean of 2.8 ± 1.0 ASMs after the second SE episode and 2.1 ± 1.2 ASMs after the first episode. Levetiracetam was the most common ASM used before admission and on discharge for SE patients.

**Conclusions:**

This retrospective, multicenter study used the mRS to demonstrate worsened outcomes of patients at consecutive SE episodes. ASM accumulations after subsequent SE episodes were registered over the study period. The study results underline the necessity for improved clinical follow-ups and outpatient care to reduce the health care burden from recurrent SE episodes.

## Key points

- We report on 120 recurrent status epilepticus episodes in 80 patients between 2011 and 2017
- The mortality rate at the second SE episode was 11.3% (9/80 patients)
- The modified Rankin Scale indicated an increased morbidity after each SE episode
- A non-refractory SE at first admission could not be identified as a protective marker for severity of following episodes
- An increased drug-load of anti-seizure medication was observed after each SE episode

## Background

Status epilepticus (SE) is a prolonged epileptic seizure that can occur with any semiological seizure form [1]. SE is a serious condition that can rapidly become a neurological emergency. In Germany, the incidence of SE is approximately 15.8 per 100,000 in population, and it occurs more often in men than women and more commonly in patients aged 60 or older [2]. SE episodes are associated with prolonged hospital stays and increased health-care costs [3–7], as well as high morbidity and short- and long-term mortality [8–10]. Furthermore, patients experiencing one episode of SE have a risk of incurring future episodes and developing chronic epilepsy [11].

Although general medical and pharmacological treatments and outcome predictors for SE have been studied extensively, little information has been documented regarding the characteristics of SE episodes occurring after an initial episode, despite reports indicating that 13–32% of all subjects are affected by recurrent SE events [2, 12, 13]. Only a few studies have specifically investigated the predictors of relapses and the prognostic role of recurrent SE [12–15]. However, information on subsequent relapses is lacking [16], despite the urgent need to identify patients who are at a high risk of recurrence [12] and adapt their clinical follow-up accordingly [13].

To address this issue, we conducted a multicenter retrospective study to provide additional information regarding repeated episodes of SE. We specifically investigated clinical characteristics and patient outcomes after subsequent episodes and compared these to the first SE episode for patients over the study period.

## Materials and methods

### Study design

This retrospective multicenter study included adult patients (aged 18 or older) with recorded episodes of non-anoxic SE between January 2011 and December 2017 at the university hospitals of Frankfurt and Marburg in the state of Hessen, Germany. Frankfurt is an urban area, whereas Marburg is a smaller university city that services its center and rural surroundings. Both offer a broad spectrum of epileptological and neurological intensive care treatments.

All pre- and in-hospital records of SE patients were reviewed and data were collected using standardized forms. Patients with incomplete clinical data were excluded.

Approval for the study was granted by the local Ethics Committees of both universities. Certain aspects of the data concerning SE treatments and outcomes were retrieved from published studies [17–22]. No sponsoring or funding was received from any company for this study. The strengthening the reporting of observational studies in epidemiology (STROBE) guidelines were followed to improve the quality of the objective documentation and reporting in this observational study [23].

### Definition of SE

The seizure type, epilepsy type, epilepsy syndrome, and SE were determined using the latest criteria of the International League Against Epilepsy (ILAE) [24–26]. These factors identify SE as a condition with abnormally prolonged seizures (after timepoint t1) that can result in long-term consequences to the patient (after timepoint t2) such as neuronal death, neuronal injury, and neuronal network alterations, depending on the type and duration of the seizure [25]. This study followed the latest definition from the ILAE, which states that SE involves a convulsive seizure lasting more than five minutes, or a nonconvulsive seizure with impaired consciousness or an absence seizure lasting greater than ten minutes [25]. Refractory status epilepticus (RSE) is defined as persisting seizures after the failure of a sufficient benzodiazepine dose as a first-line treatment and an antiseizure medication (ASM) as a second-line treatment, irrespective of time [27, 28]. Super-refractory status epilepticus (SRSE) is defined as a continuous SE episode that endures for greater than 24 h after the use of anesthetic therapy, including those cases in which seizures were controlled after anesthetic drugs were administered, but recurred once the anesthetic agent was reduced or withdrawn, this follows the definition of SRSE suggested by Simon Shorvon [29].

### Definitions of variables

The first admission to hospital due to SE during the study period was defined as the first SE episode, and subsequent episodes were numbered in order of their occurrence. Etiologies were classified as acute symptomatic, remote symptomatic, progressive symptomatic, symptomatic within new onset, and unknown (cryptogenic) [25]. We calculated the status epilepticus severity score (STESS) of each patient before treatment induction to estimate the severity of the condition [30]. The score was separated into favorable (0–3) and unfavorable (4–6) outcomes. To compare the functional status of patients before SE initiation to that at the time of discharge, we analyzed the modified Rankin Scale (mRS) using six categories (0 = no symptoms, 6 = fatal outcome), whereby categories 0–2 denoted a good functional status and categories 3–5 signified a poor functional status. We further defined the discharge destination as home, rehabilitation, care facility, other hospital, death, or other. In addition, preexisting comorbidities of the patients were collected using the Charlson Comorbidity Index (CCI) [31], which was divided into three groups (“none” = score 0; “low to moderate” = score 1–4; “multiple” = score ≥ 5). Antiseizure medication sequence, application mode, dosage, and association with onset and cessation of SE, and data on the presence of adverse events were collected. Furthermore, seizure histories or prior SEs, total lengths of hospital stays, intensive care treatments [32, 33], and intubation information were gathered. To identify a nonconvulsive SE case or the end of such a seizure, electroencephalography (EEG) was used to verify characteristic ictal patterns for SE that were validated by board certified physicians [34]. Cranial computed tomography (cCT) and cranial magnet resonance imaging (cMRI) were used as to detect acute or remote etiology correlates.

### Statistical analysis

Data were analyzed using IBM SPSS Statistics version 28.0 software (IBM Corporation, Armonk, NY, USA). Descriptive data are presented as minimum, maximum, median, mean ± standard deviation (SD), range, and percentage values. Univariate comparisons of proportions were calculated using Pearson's chi-squared test. The Mann–Whitney U test was applied for comparisons of variables of ordinal or non-normally distributed data. Two-sided *p* values less than 0.05 were considered to be significant in all statistical analyses.

## Results

During the 7-year period analyzed (2011–2017), we noted 1177 SE episodes from 1057 patients. Of these, 1057 (89.8%) were first episodes and 120 (10.2%) were recurrent episodes for 80 patients (7.6%). These patients experienced at least one relapse, while 22 (2.1%) had a second relapse and 18 (1.7%) had three or more relapses (nine patients with three, five patients with four, two patients with five, and two patients with six relapses).

### Clinical characteristics of the patients and first two SE episodes

The mean age of the patients with recurrent SE episodes at the first admission (n = 80, i.e. first episode) was 62.2 years (median 66.5; SD 19.3; range 21–91), and this mean age increased by 1.1 years at the second episode. Both episodes included 42 male (52.5%) and 38 female (47.5%) patients. At the first episode, 55 patients (68.7%) had a history of previous seizures and 19 patients (23.8%) had a prior SE episode, which was assumed to have been treated in another hospital or prior to the study period. A total of 17 patients (21.3%) had no previous comorbidities, 49 (61.2%) had low-to-moderate burdens of comorbidities, and 14 patients (17.5%) had multiple comorbidities, as evaluated by the CCI. The CCI score did not differ between both episodes (*p* = 0.612).

The time between the first and the second admission due to SE was a mean of 262.4 days (median 160 days, SD 302.4 days, range 14–1755 days).

The detailed clinical characteristics, including etiology, semiology, and diagnostics of the cohort, as well as the SE severities and patient outcomes of the first two recurrent SE episodes are shown in Table 1.

**Table 1:** Clinical characteristics, SE severities, and SE patient outcomes for the first and second episode

		First SE episode	Second SE episode	*p* value
		n = 80	n = 80	
*Clinical characteristic*				
Age	Mean ± SD in years	62.2 ± 19.2	63.3 ± 19.0	n.t.
Sex	Female/male	38/42	38/42	n.t.
Previous history of seizures	n	55 (68.7%)	80 (100%)	n.t.
Comorbidities (CCI)				0.612
None (score 0)	n	17 (21.3%)	17 (21.3%)	
Low–moderate (score 1–4)	n	49 (61.2%)	51 (63.7%)	
Multiple (score ≥ 5)	n	14 (17.5%)	12 (15.0%)	
mRS before SE onset				0.004
Score 0–2	n	36 (45.0%)	28 (35.0%)	
Score 3–5	n	44 (55.0%)	52 (65.0%)	
Etiology of SE				n.t.
Acute symptomatic	n	7 (8.8%)	9 (11.3%)	
Remote symptomatic	n	39 (48.8%)	60 (75.0%)	
Symptomatic within new onset	n	22 (27.5%)	0 (0%)	
Progressive symptomatic	n	5 (6.3%)	4 (5.0%)	
Other	n	5 (6.3%)	2 (2.5%)	
Unknown	n	2 (2.5%)	5 (6.3%)	
Semiology of SE				n.t.
Tonic–clonic SE	n	34 (42.5%)	29 (36.3%)	
Myoclonic SE	n	20 (25.0%)	22 (27.5%)	
Dyscognitive focal SE	n	15 (18.7%)	24 (30.0%)	
Absence SE	n	1 (1.3%)	2 (2.5%)	
Other or not classifiable	n	10 (12.5%)	3 (3.7%)	
Diagnostics				n.t.
cMRI	n	27 (33.8%)	20 (25.0%)	
cCT	n	71 (88.8%)	63 (78.8%)	
EEG	n	76 (95.0%)	74 (92.5%)	
*Severity of SE episodes*				
STESS at admission				0.018
Score 0–3	n	59 (73.8%)	65 (81.3%)	
Score 4–6	n	21 (26.2%)	15 (18.7%)	
SE refractoriness				0.151
NRSE	n	40 (50%)	30 (37.5%)	
RSE	n	35 (43.7%)	43 (53.7%)	
SRSE	n	5 (6.3%)	7 (8.7%)	
Intensive care unit				
Patients treated in ICU	n	70 (87.5%)	67 (83.8%)	
Length of stay (ICU)	mean ± SD in days	10.4 ± 14.3	13.2 ± 15.8	0.443
Intubation				n.t.
In context of SE treatment	n	10 (12.5%)	9 (11.3%)	
In context of airway management	n	9 (11.3%)	4 (5.0%)	
Tracheostomy	n	5 (6.3%)	6 (7.5%)	
Outcome of SE episodes				
Total length of hospital stay	mean ± SD in days	16.0 ± 14.9	15.6 ± 15.5	0.843
mRS at discharge				0.025
Score 0–2	n	25 (31.3%)	18 (22.5%)	
Score 3–5	n	55 (68.7%)	53 (66.2%)	
Score 6 (death)	n	–	9 (11.3%)	
Discharge destination				n.t.
Home	n	38 (47.5%)	31 (38.8%)	
Rehabilitation	n	20 (25.0%)	16 (20.0%)	
Other hospital	n	3 (3.8%)	3 (3.8%)	
Care facility	n	18 (22.5%)	20 (25.0%)	
Other	n	1 (1.3%)	1 (1.3%)	
Death	n	0 (0%)	9 (11.3%)	

SE, status epilepticus; mRS, modified Rankin Scale; n.t., not tested

### Severity of SE episodes

Considering the severity of SE at the first episode, 59 patients (73.8%) had a “favorable STESS-score” of 0–3, while 21 patients (26.2%) had an “unfavorable STESS-score” of 4–6. A decrease in the “unfavorable STESS-score” group was noticed with the second episode (*p* = 0.018). This was partially explained by the change in the coding of the “history of seizures” parameter toward a more favorable score for all patients.

**Fig. 1:**
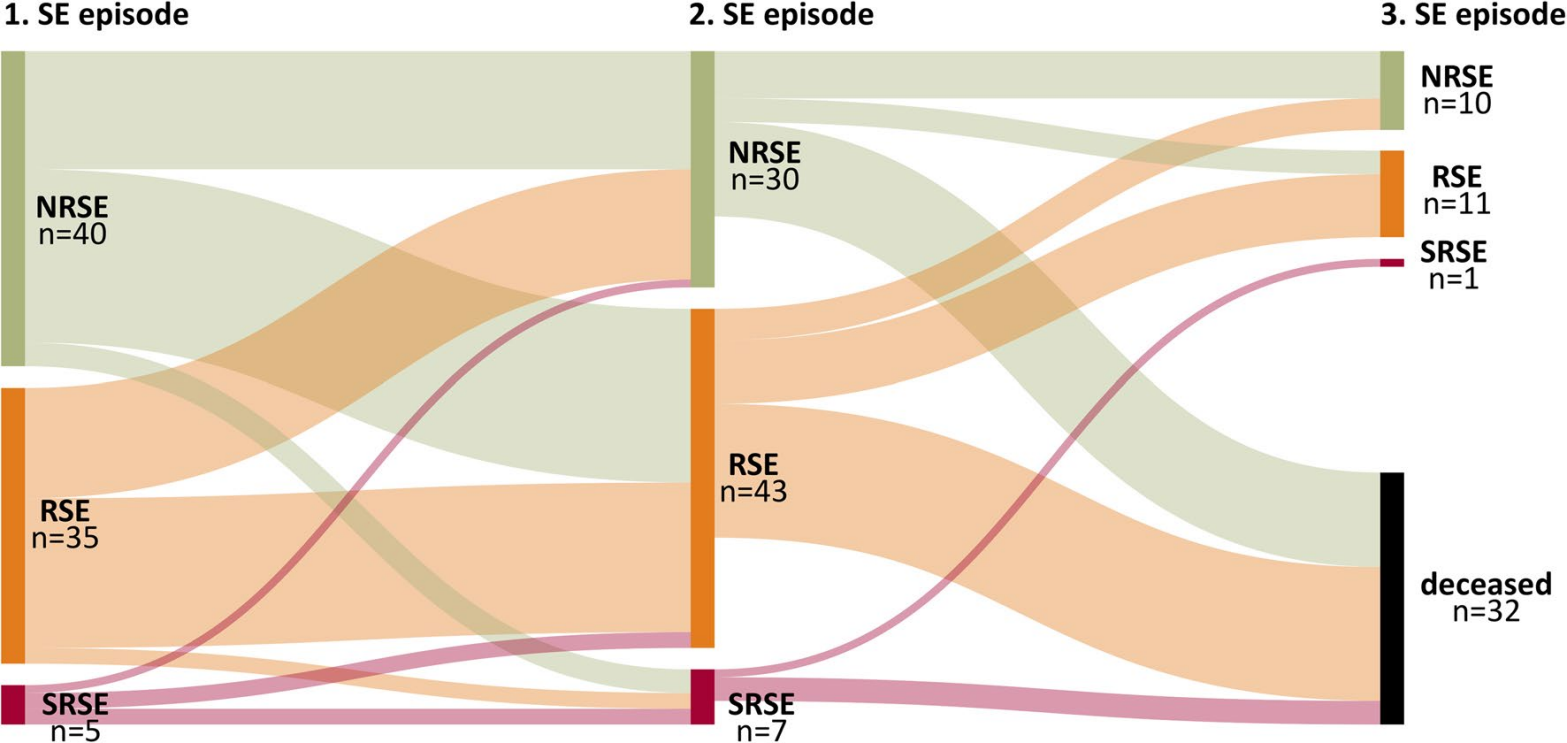
Sankey diagram of patients diagnosed with NRSE, RSE, and SRSE in their first, second, and third SE episodes (deceased refers to the 9 patients who died during the second SE episode and the 23 that died in the follow-up period)

Half of the cohort had RSE (n = 35; 43.7%) or SRSE (n = 5; 6.3%) during the first episode. An increase in RSE (n = 43; 53.7%) and SRSE (n = 7; 8.7%) was noticeable for the second event. Thus, the refractory level for individual patients changed between the episodes, as shown in Fig. 1. Consequently, the refractory level of subsequent SE episodes cannot be predicted based on the level of a former SE episode. In addition, an NRSE at the first SE episode does not provide a protective marker for a non-refractory event in subsequent episodes.

The majority of patients (n = 70; 87.5%) required intensive care treatment for a mean of 10.5 days (median 4.5, SD 14.3 days; range 1–85 days) at the first SE episode. Intubation and ventilation were used in 19 patients (23.8%). An increasing trend in the duration of ICU treatment was observed for the second SE episode (Table 1).

### Outcomes for the first two episodes

**Fig. 2:**
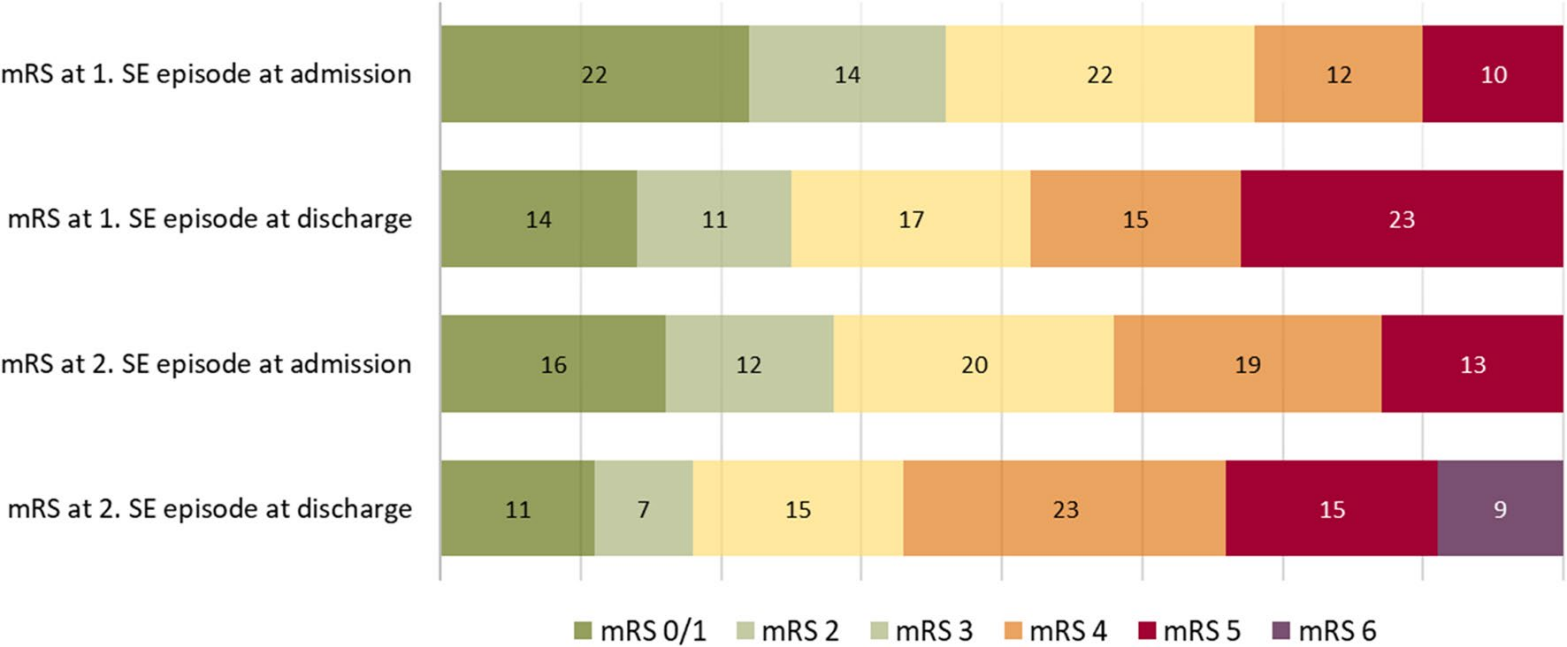
mRSs at admission and discharge for the first and second SE episodes

Comparisons were made of the functional status before SE onset and at the time of discharge using the mRS. While 36 patients (45.0%) had good functional status (scores of 0–2) at the first SE admission, 44 (55.0%) were classified with poor initial functional status (scores of 3–5). A marked decline in the functional status was noticed on hospital discharge, as 55 patients (68.7%) were identified with poor functional status at this time (*p* < 0.001). Some improvement in the functional status was noticed between the first hospitalization discharge and the second admission (*p* = 0.01). However, after the second SE episode, a clear shift to poorer outcomes and functional status was observed, as 9 patients (11.3%) died and 62 patients (77.5%) had poor outcomes (scores of 3–6) (*p* < 0.001). The number of deceased patients increased to 32 (40.0%) by the end of the follow-up in 2019. Changes in the functional status of the patients are presented in Fig. 2.

The decline in the functional status after subsequent SE episodes affected the discharge destination of the patients. After the first episode, 38 patients (47.5%) were discharged home, whereas only 31 (38.8%) were discharged home after the second episode.

### Pharmacological treatment during the first three admissions

#### First-line medication

Out-of-hospital emergency treatment with benzodiazepines (predominantly midazolam; 36/44 patients, 81.9%) was administered to 44 patients (55.0%) during the first SE episode. Emergency treatments were initiated by laypersons (7 patients, 15.9%) or ambulance service personnel (40 patients, 90.1%), and 32 patients (40.0%) began treatment at the hospital. No increase in out-of-hospital treatment was noticed during the second episode (43 patients, 53.7%, *p* = 0.873), despite the recent history of SE. Emergency treatment during the second episode was initiated by laypersons (9 patients, 20.9%) and ambulance service personnel (36 patients, 83.7%), indicating a slight increase in out-of-hospital treatments by laypersons.

Lorazepam was the most commonly used benzodiazepine in out-of-hospital and in-hospital administrations combined (first admission: n = 56; 70.0%; second admission: n = 59; 73.8%). The mean dosage of lorazepam was less than the current treatment regimen, in terms of both bolus and maintenance doses (first admission: bolus 2.22 mg ± 1.56, maintenance dose 2.97 mg ± 1.47; second admission: bolus 1.68 mg ± 0.80, maintenance dose 2.82 mg ± 1.50).

### Number of ASMs used

In total, 48 patients (60%) were taking ASMs before the first SE episode, with a mean of 1.1 ASMs per patient (SD 1.2; range 0–5); 22 patients (27.5%) were on a single ASM, 14 patients (17.5%) had two ASMs, and 12 patients (15.0%) had three or more ASMs. After the first SE episode, patients received a higher amount of ASMs when they left the hospital (mean 2.1; SD 1.2; range 1–6, *p* < 0.001), with 31 patients (38.8%) discharged with one, 24 patients (30.0%) with two, 12 patients (15.0%) with three, and 15 patients (17.5%) with four or more ASMs.

**Fig. 3:**
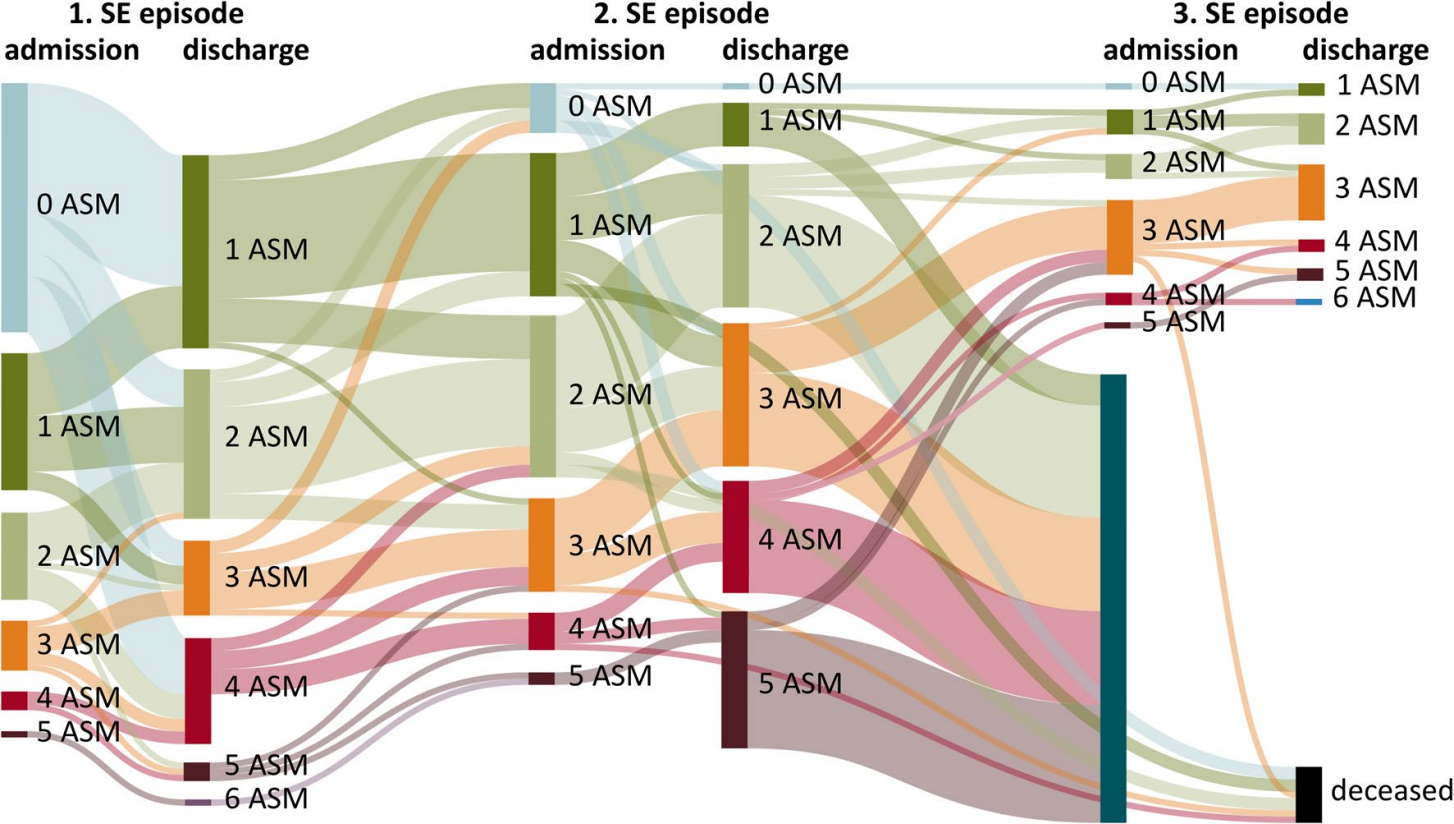
Sankey diagram showing changes in the numbers of ASMs used by patients at admission and discharge from their first, second, and third SE episode. Deceased refers to patients that died at the second SE (n = 9) or third SE episode (n = 1)

At second admission the mean number of ASMs used was 1.9 (SD 1.1; range 0–5), which was lower than that during the first discharge (*p* = 0.033). Eight patients (10.0%) were not using ASMs at this time. After the second episode, a higher amount of ASMs were administered at the time of discharge (mean 2.8; SD 1.0; range 1–5, *p* < 0.001). Subsequent SE episodes led to an accumulation of ASMs and greater numbers of patients were treated with polypharmacy, although some decreases in the number of used ASMs were noted between the episodes. Figure 3 shows the number of ASMs taken by patients at admission and at discharge from their first, second, and third SE episode.

### Use of ASMs during the first three SE episodes

Levetiracetam (n = 34; 42.5%) was the most commonly used ASM before the first SE episode, while valproate (n = 14; 17.5%), lacosamide (n = 9; 11.3%), and lamotrigine (n = 9; 11.3%) were the next most administered medications.

Medication changes were noticeable with repeated episodes. All patients were using an ASM at the time of discharge after the first episode. The medications that are available in intravenous formulations were more commonly used (especially levetiracetam, lacosamide, and valproate) than medications that are only available orally. Levetiracetam remained the most commonly used medication for all episodes, although the increase in use reached the maximum during the first episode (82.5%). By the third episode (22 patients), an increase in the use of topiramate (36.4%) and phenytoin (22.7%) was noticed, possibly relating to the treatment refractoriness of the underlying epilepsy with recurrent SE episodes. The use of ASMs before and after the first, second, and third SE episodes are shown in Table 2.

**Table 2:** Use of ASMs before and after the first, second, and third SE episodes; medications are divided into intravenously- and only orally available agents

	First SE episode (n = 80)		Second SE episode (n = 80)		Third SE episode (n = 22)	
	Before SE episode	After SE episode	Before SE episode	After SE episode *	Before SE episode	After SE episode^#^
Intravenous-available ASMs						
Levetiracetam	34 (42.5%)	66 (82.5%)	62 (77.5%)	62 (87.3%)	18 (81.8%)	17 (80.9%)
Valproate	14 (17.5%)	28 (35.0%)	26 (32.5%)	36 (50.7%)	10 (45.5%)	11 (52.4%)
Lacosamide	9 (11.3%)	26 (32.5%)	25 (31.3%)	34 (47.9%)	5 (22.7%)	10 (47.6%)
Phenytoin	1 (1.3%)	3 (3.8%)	1 (1.3%)	8 (11.3%)	2 (9.1%)	5 (23.8%)
Phenobarbital	1 (1.3%)	1 (1.3%)	1 (1.3%)	2 (2.8%)	1 (4.5%)	1 (4.8%)
Brivaracetam	–	1 (1.3%)	1 (1.3%)	2 (2.8%)	–	–
Only oral-available ASMs						
Lamotrigine	9 (11.3%)	9 (11.3%)	8 (10.0%)	11 (15.5%)	4 (18.2%)	3 (14.3%)
Topiramate	5 (6.3%)	12 (15.0%)	8 (10.0%)	15 (21.1%)	5 (22.7%)	8 (38.1%)
Zonisamide	4 (5.0%)	6 (7.5%)	6 (7.5%)	5 (7.0%)	2 (9.1%)	2 (9.5%)
Perampanel	1 (1.3%)	2 (2.5%)	1 (1.3%)	4 (5.6%)	1 (4.5%)	1 (4.8%)
No ASM intake	32 (40.0%)	–	8 (10.0%)	1 (1.4%)	1 (4.5%)	–

*Relating to 71 patients discharged alive

^#^Relating to 21 patients discharged alive

## Discussion

This retrospective, multicenter study describes the clinical characteristics of 120 recurrent SE episodes in 80 patients and provides insights into the burden of recurrent SE cases that result in increasingly poor and irreversible outcomes, which were identified by the mRS and increasing drug loads as a result of the multiple ASMs used for chronic treatment. In addition, this study reveals that a non-refractory course at the first SE episode did not exclude a refractory or super-refractory course at the second SE episode; therefore, no clinical reassurance is available for a positive outcome at the next SE episode.

In recent years, only a few research groups have focused on SE recurrence. Hesdorffer et al. [12] reported a recurrence rate of 25% in 4 years in Minnesota, while Tsetsou et al. [13] indicated a cumulative SE relapse rate of 32% in 4 years in Switzerland. A recent study by Orlandi et al. [14] showed a recurrence rate of 10.2% in a hospital-based study from 2022, in which 44 out of 430 patients relapsed. Our study results revealed that 7.6% patients experienced consecutive SE episodes. Discrepancies in recurrence rates can occur due to different study designs, inclusion criteria, and mixed populations regarding etiologies of the research groups.

Patients with recurrent SE in our study were, on average, over 60 years of age at the first episode. These results corroborate reported data, which have stated that SE occurrences were more common after the age of 60 [35, 36]. Specific causes of SE episodes such as cerebrovascular disease, anoxia, neurodegenerative disease, and brain tumors are predominantly diagnosed in older adults, which could explain this finding [35, 37]. Tsetsou et al. reported that previous strokes, brain tumors, and ASM withdrawal were the main causes of recurrent SE [13]. Furthermore, remote symptomatic etiologies, progressive symptomatic etiologies, and SRSE at the first SE episode were significantly associated with a high risk of recurrence [14]. Remote-symptomatic causes were the main etiologies in our recurrent SE cases during both admissions. The greatest risk of relapse was reported to occur during the first 6 months after the incident SE [14], which is comparative to our median 160 days between the first and second episode.

A retrospective multicenter-study from Italy indicated that SE was less likely to recur when the first episode was classified as refractory [15]. However, another study suggested that it was not refractory, but super-refractory events that were associated with a 3.9-fold increase in the risk of recurrence accompanied by an early relapse [14]. Our study showed that refractory SE was more common in second SE episodes than first SE episodes. In addition, patients with NRSE at the first SE episode presented with RSE and SRSE in subsequent episodes, which indicates that NRSE at the first SE episode was insufficient to provide a protective marker for a non-refractory event in subsequent episodes. Similarly, the refractory level of a subsequent SE episode could not be predicted by the level of a prior SE episode, since previously refractory cases could present with non-refractory SE at the next episode.

In this study, the mRS identified a clear shift to poorer outcomes and functional status with subsequent SE episodes. In addition, less patients were discharged directly after the second SE episode, which reflects that fewer patients returned to their baseline conditions. Prior studies have reported a return to the baseline in 63.0% of the cases of recurrent SE. Fatal etiologies and higher STESS scores were associated with a lack of return to the baseline, although SE recurrence itself was not directly associated [13]. Mortality increased by the second episode in our study, while Tsetsou et al. showed no reported deaths at the second or third SE recurrence [13]. However, subsequent clinical complications, such as prolonged hospitalization or intensive care, can be accompanied by an SE recurrence [38] and refraction [39], which can affect the overall outcomes of patients.

A recent German population-based study showed a 17.2% increase in rescue medication (RM) prescriptions after SE [40]. Despite a history of SE, no increase in the out-of-hospital treatment was noticed during the second episode in this study. However, it was found that medication was administered slightly more often to this group by laypersons, which indicates the availability of accessible emergency medication at home. However, improvements in clinical care are required to improve emergency care, including available fast-acting benzodiazepines in the home setting [41].

Due to the frequent occurrence of SE in patients without previously diagnosed epilepsy, the 40% of non-ASMs users in our study and 52.3% in another study [40] at the first SE admission is reasonable. Mevius et al. [40] reported up to 25.8% of non-ASM users among epilepsy patients at SE admission. As the withdrawal of ASMs is an independent risk factor for recurrent SE episodes [13] and the greatest risk for recurrence occurs in the first 6 months after SE [14], medication alterations should be thoroughly considered, especially in close relation to the SE. Our study showed a distinct accumulation of ASMs over subsequent SE episodes, which could be an indirect marker of drug resistance [15]. Gasparini et al. highlighted an association between the number of ASMs taken at the last hospitalization and the recurrence of SE [15]. In our study, ASMs that could be administered intravenously were clearly the most commonly used, with levetiracetam as the main drug administered, followed by valproate and lacosamide [42, 43]. Pharmacological treatment in the context of recurrent SE episodes should be more thoroughly investigated in future studies.

### Study limitations

This multicenter study is limited by its retrospective and non-controlled study design and includes risks in terms of methodological bias related to this type of study. However, the university hospitals in Frankfurt and Marburg offer emergency neurological treatment for a population of over one million and the patient data is systematically collected, which increases the reliability and generalizability of our results. However, this is not a population-based study, since SE patients in the area could have been admitted to other local hospitals for treatment.

Regardless of prior SE episodes in some patients, the first hospitalization during the study period was defined as the first SE episode for all patients. Considering the aim of the study was to describe the clinical characteristics of recurrent episodes, this was unlikely to cause a bias in this research setting.

## Conclusion

This study shows that the functional status of patients worsens and mortality increases with repeated SE episodes. Furthermore, a clear accumulation of ASMs occurred from recurrent SE episodes. In addition, the refractory level of a subsequent SE episode cannot be predicted by the level of a former SE episode. These results highlight the need for physicians to improve clinical follow-ups with patients and outpatient care to reduce the health care burden associated with subsequent episodes of SE.

## Data Availability

The datasets used and analyzed during the current study are available from the corresponding author on reasonable request.

## Abbreviations

ASM: Antiseizure medication
CePTER: Center for Personalized Translational Epilepsy Research
CCI: Charlson Comorbidity Index
cCT: Cranial computed tomography
cMRI: Cranial magnet resonance imaging
DRKS: Deutsches Register Klinischer Studien
EEG: Electroencephalography
ILAE: International League Against Epilepsy
mRS: Modified Rankin Scale
NRSE: Non-refractory status epilepticus
*p*: *p* Value
RSE: Refractory status epilepticus
SD: Standard deviation
SE: Status epilepticus
STESS: Status Epilepticus Severity Score
STROBE: Strengthening the Reporting of Observational Studies in Epidemiology
SRSE: Super-refractory status epilepticus
